# MiR-299-3p Inhibits Nasopharyngeal Carcinoma Cell Proliferation and Migration by Targeting MMP-2

**DOI:** 10.1155/2022/2322565

**Published:** 2022-08-25

**Authors:** Liang Tang, Mengdi Xu, Huangfeng Zhu, Yanlai Peng

**Affiliations:** ^1^Department of Otorhinolaryngology, The Affiliated Ethnic Hospital of Guangxi Medical University, Guangxi, China; ^2^Key Laboratory of Early Prevention and Treatment for Regional High Frequency Tumor (Guangxi Medical University), Ministry of Education, Guangxi, China; ^3^Guangxi Key Laboratory of Early Prevention and Treatment for Regional High Frequency Tumor, Guangxi, China; ^4^Zhejiang Baiyining Medical Laboratory Co,.Ltd., Zhejiang, China; ^5^Department of Radiation Oncology, Jiangsu Cancer Hospital, The Affiliated Cancer Hospital of Nanjing Medical University, Jiangsu Institue of Cancer Research, Nanjing, China

## Abstract

**Purpose:**

Nasopharyngeal carcinoma (NPC) is a type of squamous cell carcinoma that originated from the epithelial cells of the nose and throat, and its incidence ranks the first among head and neck tumors. However, NPC has a unique and complex etiology that is not completely understood. MiR-299-3p was discovered to be abnormally expressed in cancers. However, the involvement of miR-299-3p in the incidence and progression of nasopharyngeal cancer remains unknown.

**Methods:**

The miR-299-3p expression in nasopharyngeal cancer samples and cell lines was identified using quantitative PCR (qPCR). Nasopharyngeal cancer cells were evaluated for proliferation, migration, and invasion using MTT, colony formation assay, and Transwell invasion assay. MiRBase and TargetScan databases identified the possible miR-299-3p target genes that were confirmed using a dual-luciferase reporter analysis. Additionally, the miR-299-3p target genes were validated by Western blot, colony formation assay, and Transwell assays.

**Results:**

It was found that miR-299-3p expression was low in nasopharyngeal cancer tissues and cell lines, according to qPCR data. Cell proliferation, colony formation, and migration were considerably reduced by miR-299-3p overexpression. Furthermore, matrix metalloproteinase 2 (MMP-2) expression was regulated by miR-299-3p, whereas MMP-2 knockdown significantly inhibited the capacity of nasopharyngeal cancer cells to form colonies and migrate. Overexpression of MMP-2 substantially reduced the miR-299-3p inhibitory impact on nasopharyngeal cancer cell migration and colony formation.

**Conclusion:**

The miR-299-3p acts as a tumor suppressor gene to suppress the growth and spread of nasopharyngeal cancer by regulating MMP-2 expression. Therefore, miR-299-3p and MMP-2 could be important therapeutic targets for suppressing nasopharyngeal cancer growth and metastasis.

## 1. Introduction

Nasopharyngeal carcinoma (NPC) originates from the nasopharyngeal epithelium and has a high degree of malignancy, invasiveness, and distant metastases. It has a high incidence and ranks at the top among malignant tumors in the head and neck [1, 2]. It is believed that the main pathogenic factors of nasopharyngeal carcinoma mainly include heredity, environmental factors, and EB virus infection. Epidemiological surveys indicate that the incidence of nasopharyngeal carcinoma shows a significant upward trend. The clinical treatment of nasopharyngeal carcinoma mainly adopts radiotherapy and chemotherapy [3]. Treatment effectiveness has increased; however, the prognoses remain dismal and patients may develop distant metastases within four years. At present, radiotherapy is recognized as one of the effective treatment methods for nasopharyngeal carcinoma, but the effect of radiotherapy in clinical practice is not satisfactory, and recurrence and metastasis often occur [3, 4]. In addition, TNM staging of nasopharyngeal carcinoma is closely related to the treatment effect and prognosis. It is easy to treat in the early stage and has a poor prognosis in the advanced stage. Therefore, the study on biomarkers and features of nasopharyngeal cancer has tremendous value for its treatment.

MicroRNAs (miRNAs) are noncoding short RNA molecules of 18–24 nucleotides and are found in a wide variety of organisms, including viruses, plants, and animals. It is involved in various biological processes such as apoptosis [5, 6]. Previous studies have shown that the role of miRNA is to inhibit gene expression at a transcriptional level via incomplete complementary pairing with multiple target genes [5]. Numerous studies have shown a link between cancer onset and deregulation of miRNA expression. Therefore, miRNAs are of great significance for the early diagnosis and treatment of cancers [7]. For example, miRNA-21 may act as a biomarker in peripheral blood for prostate cancer progression [8]. MiRNAs in peripheral blood are useful diagnostic biomarkers for nonalcoholic steatohepatitis [9]. As a biomarker for predicting prognosis in gastric cancer, miRNA-194 may be utilized to target CCND1 and limit gastric cancer cell invasion and propagation [10]. In addition, multiple miRNAs take part in the development, differentiation, and proliferation of nasopharyngeal carcinoma, affecting the prognosis of patients [11]. It has been shown that overexpression of the miR-30a-5p may decrease the development, differentiation, and invasion of nasopharyngeal carcinoma cells. *In vitro*, inhibiting NUCB2 decreases nasopharyngeal cancer cell invasion and propagation [12]. The abnormal expression of miR-299-3p was associated with tumor invasion and metastasis, and miR-299-3p may affect tumor occurrence and development by regulating target genes [13, 14]. However, the miR-299-3p expression and its role in nasopharyngeal carcinoma have not been reported. This work investigated the expression of miR-299-3p in NPC tissues and elucidated its regulatory role.

## 2. Material and Methods

### 2.1. Tissue Sample Collection

For this study, 27 patients diagnosed with nasopharyngeal cancer between 2020 and 2021 were included. The patients were aged 36–71 years old, with an average age of 49 years. The patients' cancerous tissue and paracancerous normal tissue (more than 3 cm away from the tumor edge) were collected prior to chemotherapy. The collected tissues were placed in liquid nitrogen for cryopreservation. Samples used in this study were approved by the Medical Ethics Committee of The Affiliated Ethnic Hospital of Guangxi Medical University. Inclusion criteria are as follows: (1) all cases were biopsied by electronic nasopharyngoscope, and were pathologically diagnosed as primary nasopharyngeal carcinoma; (2) they had complete clinical diagnosis and treatment data; (3) the patients had no serious heart, kidney, lung, or liver disease and other systemic diseases; (4) family members and patients were aware of the research process, purpose, and significance, and agreed to participate.

### 2.2. Cell Culture and Transfection

The China Center of Type Culture Collection provided nasopharyngeal epithelial cells (NP69) and nasopharyngeal cancer cell lines CNE-1, CNE-2, HNE-1, and C666-1. The cells were grown with 10% fetal bovine serum in RPMI1640 media incubated at 37°C with 5% CO_2_. The miR-299-3p mimics, control mimics, pcDNA-vector, pcDNA matrix metalloproteinase 2 (MMP-2), shRNA-control, and shRNA-MMP-2 were synthesized and constructed by Guangzhou Ribo Biotechnology Co., Ltd. The cells were then transfected and all transfected cells were grown in a mixture of 1% dual antibiotics (penicillin and streptomycin) and 10% bovine serum at a constant temperature with 5% CO_2_ at 37°C. The medium was replenished every three days. Experiments were carried out after the cells grew to 80%–90% confluence, and total cellular RNA was extracted 48 hours after transfection.

### 2.3. qPCR Detection

The extraction of total RNA from tissues and cells was carried out following the Trizol method and the RNA concentration was determined using an ultra-micro spectrophotometer. The cDNA was reversely transcribed using a template of 100 ng total RNA. The reaction temperatures were 37°C and 98°C for 15 and 5 min, respectively. Furthermore, the PCR reaction was carried out according to the instructions of the PCR kit. From the acquired data, the mRNA expression was computed using 2^−ΔΔCT^. The miR-299-3p (Forward, 5′-TTCAGTGTAAACATCCTCGACTG-3′; Reverse, 5′-TGGCAATGTCGTGGAGTCG-3′) and U6 primer sequences were referenced in [13, 15], and the primers for MMP-2 and GAPDH were in reference in [16]. Eventually, the experiment was repeated three times.

### 2.4. MTT Assay for Cell Viability

A single-cell solution containing 10% fetal calf serum was prepared by transfecting miR-299-3p mimics and control mimics. In 96-well plates having a 200 *μ*l volume for each well, 2.0 × 10^3^ cells were seeded. After that, the plates were cultured for various periods. Additionally, 10 *μ*l of MTT solution (5 mg/ml, produced with PBS, pH = 7) was added and incubated for 4 hours at 37°C before being read on a microplate reader after 24, 48, 72, and 96 hours.

### 2.5. Clonogenic Assay

A single-cell suspension generated from nasopharyngeal cancer cells in the logarithmic growth phase was obtained. These cells were seeded onto 6-well plates, with 700 cells per well, after transfection. One-third of the media was replenished every three days while the cells were cultured at 37°C and 5% CO_2_. After three weeks of continuous culture, cells were fixed in 4% paraformaldehyde followed by 15 minutes of staining in 0.1% crystal violet solution and then air dried. After that, images were taken and statistical analysis was performed.

### 2.6. Transwell Detection Assay

The invasive ability of nasopharyngeal carcinoma CNE-1 cells was measured by Transwell assay. The experiment was split into two groups: experimental and control groups. CNE-1 cells (had an adjusted density of 5 × 10^4^/ml) transfected with miRNA-299-3p-mimics were inoculated in the top chamber with serum-free media in the experimental group. CNE-1 cells transfected with mRNA-299-3p control were seeded in 24-well plates at the same concentration. The bottom chamber was added with a 15% FBS medium. The cells from the top chamber were wiped via cotton ball after 48 hours. Subsequently, 4% paraformaldehyde and crystal violet were used to fix and stain the cells on the bottom side of the membrane. A light microscope was used to count the invading cells (10×, 5 randomly selected fields per well).

### 2.7. Dual-Luciferase Reporter Assay

The luciferase reporter vector and miRNA-299-3p mimics were cotransfected into nasopharyngeal cancer cells in order to investigate the miRNA-299-3p binding to the 3′UTR of MMP-2. After 36 hours of transfection, cells containing MMP-2 Mut and MMP-2 Wt were collected, and miRNA-299-3p mimics as well as control mimics were transfected. For this experiment, the luciferase activity of individual cells was measured using a luciferase activity detection kit, and the following formula was used to calculate the results:

luciferase activity = firefly-luciferase activity value/renilla-luciferase activity value.

### 2.8. Western Blotting Analysis

The total proteins of cells were collected, and their concentration was detected by the BCA method, and 10% SDS-PAGE separation gel was prepared for sample separation. Wet transfer electrophoresis was used to transfer 60 *μ*g of protein from each well to a PVDF membrane. After blocking the membrane with 5% fat-free milk for two hours, the membrane was washed for five minutes with TBST. Then, it was added with diluted 1 : 500 TBST primary antibody (ZEB2) and incubated at 4°C overnight. TBS was used to rinse the sample for 5 minutes, then a 1 : 2 000 secondary antibody was introduced and incubated for 2 hours at room temperature. TBST was used to wash the membrane for ECL luminescence imaging for 5 minutes. The experimental results were recorded and the experiment was repeated thrice.

### 2.9. Statistical Analysis

All the experiments were performed thrice in order to ensure accuracy. The mean and standard deviation are shown in the bar and curve graphs. ANOVA was used to examine the data. The statistical analysis was performed via SPSS 20.0. Statistical significance was set at a *P* value <0.05.

## 3. Result

### 3.1. The MiR-299-3p Was Expressed at Low Level in Nasopharyngeal Carcinoma Tissues and Cell Lines

**Figure 1:**
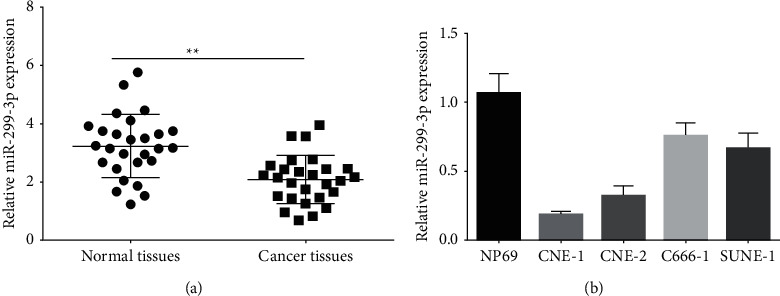
qPCR detection of miR-299-3p expression in nasopharyngeal cancer tissues (a) and cell lines (b). ^*∗∗*^*P* < 0.01.

The miR-299-3p expression levels from 27 nasopharyngeal paracancerous and paracarcinoma (normal) tissues were detected by qPCR. MiR-299-3p expression in nasopharyngeal cancer tissues was lower than that in paracancerous normal tissues as shown in Figure 1(a). Several nasopharyngeal cancer cell lines were found to express miR-299-3p. Consequently, it was determined that miR-299-3p expression in nasopharyngeal cancer cell lines was reduced compared to normal nasopharyngeal epithelial cells (NP69), with the lowest expression in CNE-1 cells (Figure 1(b)). Therefore, CNE-1 was selected as the cell line for subsequent experiments.

### 3.2. MiR-299-3p Overexpression Significantly Prevented the Nasopharyngeal Carcinoma Cell Proliferation and Migration

**Figure 2:**
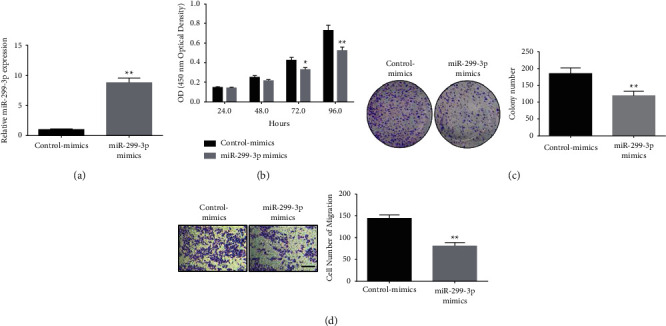
MiR-299-3p overexpression inhibits nasopharyngeal cancer cell migration and growth. (a) qPCR detection of intracellular miR-299-3p mimics expression in CNE-1 cells; (b) MTT assay detecting the effect of transfected miR-299-3p mimics on the CNE-1 cells proliferation ability; (c) colony formation assay to detect the effect of transfected miR-299-3p mimics on the CNE-1 cells clonogenic ability; (d) Transwell detection of transfection miR-299-3p mimics on CNE-1 cell migratory ability. ^*∗∗*^*P* < 0.01, ^*∗*^*P* < 0.05, scale bar = 100 *μ*m.

The miR-299-3p mimic was synthesized and then transfected into CNE-1 cells. qPCR was used to measure the expression efficiency. Cellular miR-299-3p expression was increased via miR-299-3p mimics (Figure 2(a)). Moreover, the MTT and colony formation assays were used to examine the impact of miR-299-3p overexpression on nasopharyngeal cancer cell growth. Transfection with miR-299-3p mimics substantially decreased cell proliferation and colony formation compared to the control group (Figures 2(b) and 2(c)). The miR-299-3p mimic substantially suppressed the migration ability of cells compared to the control group (Figure 2(d)).

### 3.3. MiR-299-3p Regulated MMP-2 Expression in Nasopharyngeal Carcinoma Cells

**Figure 3:**
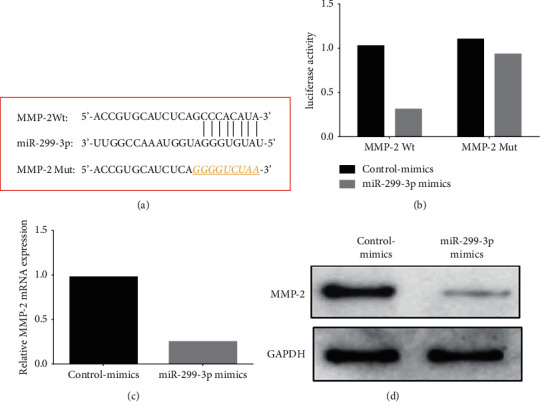
MiR-299-3p regulates MMP-2 expression in nasopharyngeal carcinoma cells; (a) binding sites and mutation sites of miR-299-3p and MMP-2; (b) luciferase reporter assay detecting the miR-299-3p and MMP-2 binding activity; (c) qPCR to determine the impact of miR-299-3p mimics on intracellular MMP-2; (d) Western blot was used to determine the impact of miR-299-3p mimics on intracellular MMP-2. ^*∗∗*^*P* < 0.01.

TargetScan analysis tool identified the binding sites between MMP-2 and miR-299-3p (Figure 3(a)). For MMP-2 Wt, the miR-299-3p mimic dramatically reduced luciferase activity, whereas the control-mimics group had no such effect in MMP-2 mutants. There was no difference in luciferase activity between the miR-299-3p and the control-mimics groups (Figure 3(b)). We used qPCR and Western blot to further investigate the effect of miR-299-3p mimics on MMP-2 expression in cells. Using miR-299-3p mimics, nasopharyngeal cancer cells showed substantial decreases in MMP-2 mRNA and protein expression levels.

### 3.4. Knockdown of MMP-2 Expression Prevented the Migration and Proliferation of Nasopharyngeal Carcinoma Cells

**Figure 4:**
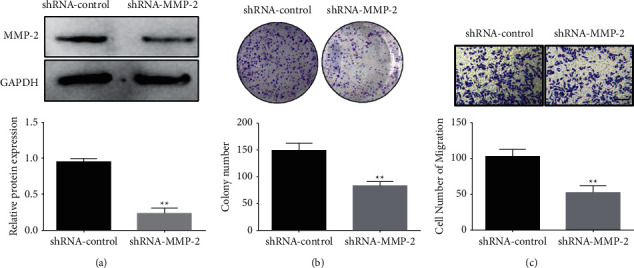
Knocking down MMP-2 expression inhibits nasopharyngeal cancer cell growth and migration. (a) qPCR to determine the level of intracellular MMP-2 expression, (b) colony formation assay showing the effect of MMP-2 knockdown on CNE-1 cell's capacity to generate colonies, and (c) Transwell assay determining the influence of MMP-2 knockdown on CNE-1 cells ability to migrate. ^*∗∗*^*P* < 0.01, ^*∗*^*P* < 0.05. Scale bar = 100 *μ*m.

The lentiviral knockdown vector (shRNA-MMP-2) of MMP-2 was synthesized and transfected into CNE-1 cells. Knockdown of the vector (shRNA-MMP-2) significantly inhibited the expression level of intracellular MMP-2 (Figure 4(a)). The colony formation test was performed to investigate the impact of MMP-2 knockdown on nasopharyngeal cancer cell growth. According to the findings, lentiviral MMP-2 knockdown vectors (shRNA-MMP-2) substantially inhibited cell colony formation compared to that of the control group (shRNA-control) (Figure 4(b)). Moreover, compared to the control group, the suppression of cell migration was shown to be significantly reduced in transfected miR-299-3p mimics cells (shRNA-MMP-2 lentiviral knockdown vector) (Figure 4(c)).

### 3.5. MiR-299-3p inhibited Migration and Proliferation of Nasopharyngeal Carcinoma Cells via Regulating MMP-2 Expression

**Figure 5:**
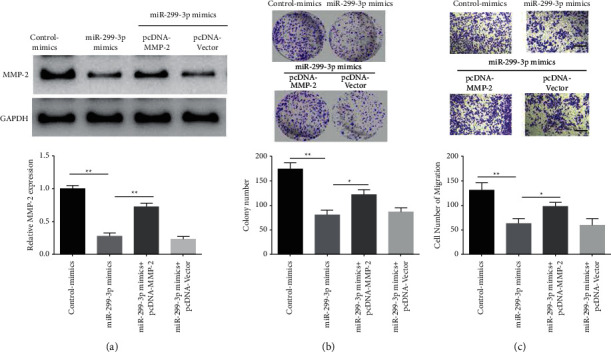
MiR-299-3p inhibits the migration and proliferation of nasopharyngeal carcinoma cells via regulating MMP-2 expression. (a) Western blot on the MMP-2 expression regulated by miR-299-3p, (b) miR-299-3p inhibited the nasopharyngeal cancer cells proliferation by regulating the MMP-2 expression, and (c) miR-299-3p inhibited the nasopharyngeal cancer cells migration via regulating MMP-2 expression, ^*∗∗*^*P* < 0.01, ^*∗*^*P* < 0.05.

We further evaluated whether miR-299-3p decreased nasopharyngeal cancer cell proliferation and migration through modulating the MMP-2 gene expression. It was found that mimics of miR-299-3p substantially inhibited intracellular MMP-2 expression compared to the control-mimics group, whereas overexpression of MMP-2 (pcDNA-MMP-2) in the mimics group of miR-299-3p considerably reversed the inhibition of MMP-2 expression (Figure 5(a)). Additionally, miR-299-3p mimics transfection considerably inhibited cell colony formation compared to the control group, whereas overexpression of MMP-2 (pcDNA-MMP-2) in the miR-299-3p mimics dramatically prevented the miR-299-3p inhibitory impact on cell colonies in the mimics group (Figure 5(b)). MiR-299-3p mimics transfection significantly repressed cell migration compared to the control group, however, overexpression of MMP-2 (pcDNA-MMP-2) in the mimics significantly prevented miR-299-inhibitory impact on cell migration (Figure 5(c)).

## 4. Discussion

MiRNAs are single-stranded noncoding RNAs with short lengths and highly conserved sequences that operate on the 3′-end noncoding regions of mRNA to control the expression of target genes [17, 18]. miRNAs are abnormally expressed in various cancers, and play different physiological regulatory functions as tumor-promoting or tumor-suppressing genes. For example, miRNA-221-5p is highly expressed in bladder cancer, which is related to lymph node metastasis, and distant metastasis regulates E-cadherin expression to promote bladder cancer cell invasion and migration, leading to poor prognosis of patients [19]. In gastric cancer tissues, miR-299-3p is underexpressed; that is by blocking its expression it may increase cancer cell invasion, whereas overexpressing miR-299-3p significantly reduces cell invasion [14]. According to the findings of the current study, nasopharyngeal carcinoma tissues and cell lines have low miR-299-3p expression, while its overexpression may greatly reduce the propagation and migration of cancer cells. MiR-299-3p was observed to decrease MMP-2 expression in studies of its molecular mechanism.

The tumor suppressor gene miR-299-3p has been shown to be underexpressed in many tumor tissues, including lung cancer, gastric cancer, liver cancer, and oral squamous cell carcinoma [13, 15, 20, 21]. For instance, miR-299-3p expression is decreased in cancerous liver tissue, whereas, its overexpression suppresses liver cancer cell migration, invasion, and proliferation. Since miR-299-3p targets SIRT5, it prevents liver cancer cells from migrating, invading, or proliferating. miR-299-3p could be prognostic indicators and therapeutic targets for liver cancer [15]. Previous studies have shown that nasopharyngeal cancer tissues and cell lines are miR-299-3p deficient. However, its overexpression promoted cell propagation and invasion. Because target genes are regulated via miRNAs by acting on the 3′-end noncoding regions of mRNA encoding genes, further online bioinformatics prediction, luciferase reporter gene, and qPCR detection showed that miR-299-3p regulates the MMP-2 expression through binding in nasopharyngeal carcinoma cells.

Extracellular matrix proteins are degraded and remodeled by a family of zinc-dependent proteolytic enzymes known as matrix metalloproteinases (MMPs) [22]. Nasopharyngeal cancer tissues had an MMP-2 activity level much greater than normal nasopharyngeal tissues, and the clinical stage of nasopharyngeal carcinoma was strongly correlated with MMPs activity [23, 24]. MMP-2 suppression may prevent the invasion and spread of nasal cancer cells, such as *Rubus idaeus*, which inhibits the invasion and migration of human nasal cancer cells by regulating MMP-2 levels [25]. The MMP-2 production and cancer cell invasion in human nasopharyngeal carcinoma may be inhibited by salvianolic acid A via ERK signaling [26]. The findings of this study demonstrated that nasopharyngeal cancer cell proliferation and migration could be inhibited by knocking down MMP-2 expression. Additional analysis revealed that overexpression of miR-299-3p inhibited the overexpression of MMP-2 in nasopharyngeal carcinoma cells whereas MMP-2 overexpression halts the miR-299-3p impact on the migratory and propagation inhibition of nasopharyngeal carcinoma cells.

In conclusion, the findings of this study show that miR-299-3p expression is decreased in nasopharyngeal cancer tissues and cell lines. Overexpression of miR-299-3p inhibits cell proliferation and migration. MiR-299-3p regulates MMP-2 expression. MMP-2 knockdown drastically reduced nasopharyngeal cancer cell growth and migration, while overexpression of MMP-2 prevented the inhibitory impact of miR-299-3p overexpression on nasopharyngeal cancer cells migration and propagation. These findings indicate that miR-299-3p is an important target for reducing the development and spread of nasopharyngeal cancer, with therapeutic implications.

## Data Availability

All experimental data used to support the findings of this study are available from the corresponding author upon request.
